# Feasibility and impact of providing feedback to vaccinating medical clinics: evaluating a public health intervention

**DOI:** 10.1186/1471-2458-10-750

**Published:** 2010-12-03

**Authors:** Nicholas Brousseau, Chantal Sauvageau, Manale Ouakki, Diane Audet, Marilou Kiely, Colette Couture, Alain Paré, Geneviève Deceuninck

**Affiliations:** 1 Institut national de santé publique du Québec, Québec, Canada; 2 Centre de recherche du CHUL-CHUQ, Centre Hospitalier Universitaire de Québec, Québec, Canada; 3 Direction régionale de santé publique de la Capitale-Nationale, Québec, Canada; 4 Université Laval, Québec, Canada

## Abstract

**Background:**

Vaccine coverage (VC) at a given age is a widely-used indicator for measuring the performance of vaccination programs. However, there is increasing data suggesting that measuring delays in administering vaccines complements the measure of VC. Providing feedback to vaccinators is recognized as an effective strategy for improving vaccine coverage, but its implementation has not been widely documented in Canada. The objective of this study was to evaluate the feasibility of providing personalized feedback to vaccinators and its impact on vaccination delays (VD).

**Methods:**

In April and May 2008, a one-hour personalized feedback session was provided to health professionals in vaccinating medical clinics in the Quebec City region. VD for vaccines administered at two and twelve months of age were presented. Data from the regional vaccination registry were analysed for participating clinics. Two 12-month periods before and after the intervention were compared, namely from April 1^st^, 2007 to March 31^st^, 2008 and from June 1^st^, 2008 to May 31^st^, 2009.

**Results:**

Ten medical clinics out of the twelve approached (83%), representing more than 2500 vaccinated children, participated in the project. Preparing and conducting the feedback involved 20 hours of work and expenses of $1000 per clinic. Based on a delay of one month, 94% of first doses of DTaP-Polio-Hib and 77% of meningococcal vaccine doses respected the vaccination schedule both before and after the intervention. Following the feedback, respect of the vaccination schedule increased for vaccines planned at 12 months for the four clinics that had modified their vaccination practices related to multiple injections (depending on the clinic, VD decreased by 24.4%, 32.0%, 40.2% and 44.6% respectively, p < 0.001 for all comparisons).

**Conclusions:**

The present study shows that it is feasible to provide personalized feedback to vaccinating clinics. While it may have encouraged positive changes in practice concerning multiple injections, this intervention on its own did not impact vaccination delays of the clinics visited. It is possible that feedback integrated into other types of effective interventions and sustained over time may have more impact on VD.

## Background

Child vaccination is one of the most effective interventions in public health [1,2]. Vaccine coverage (VC) at a given age is a widely-used indicator for measuring the performance of vaccination programs [3]. However, there is increasing data suggesting that measuring delays in age-appropriate vaccination complements the measure of VC [4-6]. Vaccination delays (VD) can, independently of VC, increase the burden of certain childhood diseases such as pertussis and measles [7,8]. Moreover, a delay in the administration of vaccines scheduled for the age of two months is associated with poor VC at two years [3,9,10].

Across Canada, a consensus has been reached on the definition of VD, namely one month after the due date on the vaccination schedule [11]. This definition is in line with the principal studies on the subject [4,6,12-15]. However, since 2006, the Quebec Ministry of Health and Social Services has compiled VD noted in Health and Social Service Centres (*Centres de santé et de services sociaux *or CSSS) based on a delay of one week following the recommended age [16]. This tracking of VD is not carried out in private medical clinics, which administer about half of the vaccines for infants in the province of Quebec [3,17].

In 2008, in Quebec, using a one-month delay definition, only 21% of children received all the vaccines scheduled before the age of two years within the recommended period [3].

Providing feedback to vaccinators is one of the effective strategies for improving VC [18-24]. It involves presenting clinicians with their performance data, with or without recommendations [25]. This intervention is carried out following data collection on clinicians' performance (audit). Certain studies have shown that feedback may help reduce VD and missed vaccination opportunities [18,26]. On the other hand, the impact and feasibility of providing feedback to vaccinating medical clinics have not been documented extensively in a Canadian context [27,28].

The main objective of this study was to evaluate the feasibility and the impact on VD of providing personalized feedback to medical clinics that vaccinate infants in the Quebec City region.

## Methods

### Participants

The twelve medical clinics that had administered the largest number of doses of DTaP-Polio-Hib vaccine in 2007 in the Quebec City region were approached to participate in the feedback project. The vaccination registry (VAXIN), which compiles all doses administered in the Quebec City region, enabled to identify these clinics and to evaluate vaccination delays.

### Intervention

In April and May 2008, a one-hour feedback session, led by a physician and a public health nurse, was carried out with the physicians, nurses and secretaries in each participating medical clinic. This feedback dealt with VD for infants at the clinic for the year 2007. Data on the proportion of doses administered without delay were presented for the first doses of three vaccines (DTaP-Polio-Hib, pneumococcal and meningococcal). Vaccination delays for each clinic were presented both in terms of the Quebec standard (one week) and the proposed Canadian standard (one month). Graphs showing the cumulative percentage of children vaccinated according to age were also presented for vaccines scheduled at 2 and 12 months, including measles, mumps and rubella vaccine (MMR). During preparation of the feedback, it became clear that certain clinics were not administering vaccines scheduled at one year during a single visit. Consequently, information on the importance of multiple injections was transmitted to these medical clinics. Ethical approval was obtained from the *Comité d'éthique de la recherche du CHUQ - Centre hospitalier de l'Université Laval *(project C10-11-101).

### Questionnaire

At the end of each feedback session, a questionnaire on the organizational characteristics of the clinic in terms of vaccination was completed on site by a nurse or a secretary. One year following the feedback session (July 2009), the same questionnaire was completed by telephone and, when possible, with the same person.

### Feasibility

The data related to the feasibility of the intervention, i.e., the material, human and financial resources required, were compiled.

### Analyses of vaccination delays

In order to measure the impact of the feedback on delays associated with vaccines (1^st ^dose of DTaP-Polio-Hib, 1^st ^dose of pneumococcal, meningococcal and 1^st ^dose of MMR), data from the regional vaccination registry were analyzed for the participating clinics. Two 12-month periods before and after the intervention were compared, namely from April 1^st^, 2007 to March 31^st^, 2008 and from June 1^st^, 2008 to May 31^st^, 2009. Two indicators of delay were considered: one week and one month after the due date on the vaccination schedule. Proportion comparisons were carried out using the chi-square test. The distributions of ages at vaccination, before and after the intervention, were compared using the Wilcoxon test. The analyses were carried out using the SAS 9.1 software (SAS Institute, Cary, NC).

## Results

### Participation

A total of ten clinics (83%), representing more than 2500 vaccinated children, agreed to participate in the feedback project. Out of the 206 physicians, nurses and secretaries working in these ten clinics, 106 participated in the feedback process (51%).

### Organizational characteristics of the medical clinics

Table 1 summarizes the organizational characteristics of the clinics visited and the changes observed between the ends of each of the two observation periods. Among other characteristics, it was noted that four clinics changed their practices concerning multiple injections. For two of these four clinics, responsibilities concerning vaccination were given to nurses. Since 2002, in the province of Quebec, nurses have the possibility to vaccinate as set out in the Public Health Act [29].

**Table 1:** Characteristics of clinics at the end of the second observation period and changes observed

Clinic	Organizational characteristics (X = yes)						
	**If delay noted:**						
					
	**Child seen very rapidly (otherwise, child referred)**	**Possibility of vaccinating a child without appointment**	**Possibility of extending clinic hours**	**Possibility of making an appointment with a nurse only**	**Multiple injections at 12 months encouraged**	**Possibility of making an appointment on site**	**Telephone reminder before an appointment**

**01**					X	X	
**02**					X		X
**03**	X	X		X	X		
**04**	X	X		X	X	X	
**05**	X	X	X	X^a, c^	X^b^	X	X
**06**	X	X	X	X^c^	X^b^	X	
**07**					X		
**08**	X					X	
**09**					X^b^	X	X
**10**	X	X			X^b^	X	

^a ^During the second observation period, hiring of two nurses who vaccinate on an appointment basis.

^b ^Unlike the end of the first observation period, multiple injections are now encouraged.

^c ^During the second observation period, nurses began vaccinating children at 12 months on an appointment basis.

### Vaccination delays

During the second observation period, 94% of the first doses of DTaP-Polio-Hib and pneumococcal vaccines were received within the one-month delay (Table 2). This proportion was 77% for meningococcal vaccine and 75% for MMR (Table 3). When a delay of one week after the scheduled vaccination date was applied, the proportion of doses received without delay was 56% for DTaP-Polio-Hib and pneumococcal vaccines, 34% for meningococcal vaccine and 33% for MMR.

**Table 2:** Doses of vaccines administered without delay before and after feedback for the 10 clinics

Vaccines planned at 2 months of age and indicator	Proportion without delay Before feedback (2007-2008)	Proportion without delay After feedback (2008-2009)	Difference	p
1^st ^DTaP-Polio-Hib	Doses = 3297	Doses = 3519		
				
1 week	64.8%	55.9%	- 8.9%	< 0.001
1 month	93.6%	93.6%	0.0%	0.970
				
1^st ^Pneumococcal	Doses = 3272	Doses = 3504		
				
1 week	65.0%	56.0%	- 9.0%	< 0.001
1 month	94.0%	93.8%	- 0.3%	0.656

**Table 3:** Doses of vaccines administered without delay before and after feedback for the 10 clinics

Vaccines planned at 12 months of age and indicator	Proportion without delay Before feedback (2007-2008)	Proportion without delay After feedback (2008-2009)	Difference	p
Meningococcal	Doses = 2787	Doses = 2870		
				
1 week	36.9%	34.2%	- 2.7%	0.036
1 month	77.0%	77.0%	+ 0.0%	0.973
				
1^st ^MMR	Doses = 2858	Doses = 2941		
				
1 week	33.9%	33.2%	- 0.8%	0.525
1 month	72.7%	74.9%	+ 2.2%	0.053

Few changes were observed before and after the feedback (Tables 2 and 3). Using the one-month delay, no statistically significant difference in VD was found between the two periods. Using the one-week delay, a statistically significant increase in VD was observed for DTaP-Polio-Hib (8.9%, p < 0.001), pneumococcal (9.0%, p < 0.001) and meningococcal (2.7%, p = 0.036) vaccines.

The median age at vaccination increased in a statistically significant manner for DTaP-Polio-Hib and pneumococcal vaccines (+ 2 days, p < 0.0001). For all vaccines, the difference in median age at vaccination before and after the feedback was nevertheless very small, consistently less than 3 days (Table 4).

**Table 4:** Median age at vaccination before and after feedback for the 10 clinics

	Before feedback (2007-2008)		After feedback (2008-2009)			
**Vaccine**	**Doses** **administered**	**Median age** **(months)**	**Doses administered**	**Median age** **(months)**	**Difference** **(days)**	**p**

1^st ^DTaP-Polio-Hib	3297	2.14	3519	2.20	+ 2	< 0.0001
1^st ^Pneumococcal	3272	2.14	3504	2.20	+ 2	< 0.0001
Meningococcal	2787	12.39	2870	12.42	+ 1	0.07
1^st ^MMR	2858	12.45	2941	12.45	0	0.65

**Figure 1:**
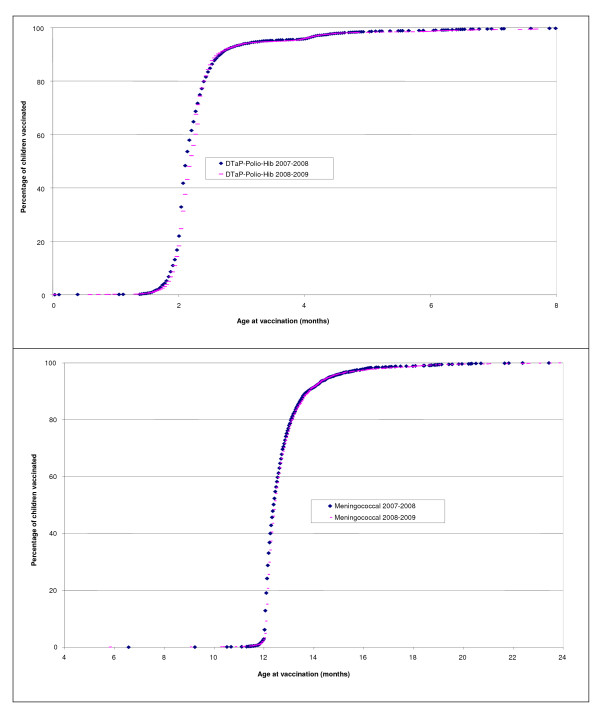
**Cumulative percentage of children vaccinated in the 10 clinics before and after the feedback intervention**.

As can be seen in Figure 1, age at vaccination for DTaP-Polio-Hib and meningococcal vaccines for the 2008-2009 period was comparable to that for the 2007-2008 period, with both curves being superimposed.

**Figure 2:**
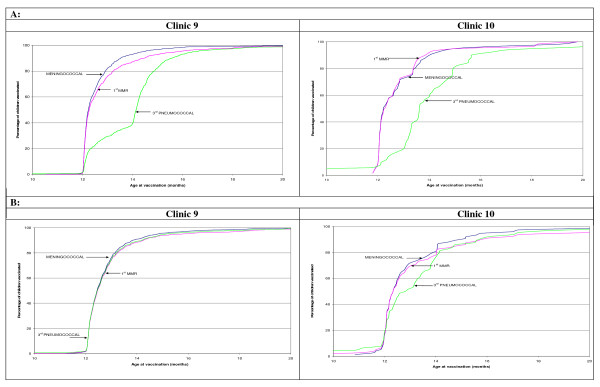
**Cumulative percentage of children vaccinated before (A) and after (B) feedback for two clinics**.

For the four clinics that began the practice of multiple injections in 2008-2009, a significant decrease in VD was observed for vaccines scheduled at 12 months. For the third dose of the pneumococcal vaccine (given at 12 months), the proportion of infants immunized within a one-month delay increased from 19.6% to 51.6% (p < 0,001) for clinic 10 and from 29.3% to 73.9% (p < 0,001) for clinic 09. The proportion of infants immunized without delay for the first dose of MMR increased from 27.4% to 67.6% (p < 0,001) for clinic 05. Finally, the proportion of infants immunized without delay for the meningococcal vaccine increased from 56.5% to 80.9% (p < 0,001) for clinic 06. Figure 2 (a and b) illustrates this situation for two of the four clinics.

### Feasibility

The preparation and completion of the feedback sessions required, in terms of human resources, the contribution of a physician (42 hours), two nurses (48 hours), a statistician (42 hours) and a research officer (74 hours). The cost of preparing and carrying out the meetings in the ten clinics (professional hours, materials, travel and cold meals served) was estimated at $10,000.

## Discussion

The feedback process conducted in 10 of the 12 main vaccinating clinics in the Quebec City region did not decrease overall vaccination delays. A high proportion of the DTaP-Polio-Hib and pneumococcal vaccines were administered before the age of three months (94%), both before and after the intervention. The proportion of doses administered without delay was lower for meningococcal (77%) and MMR (73-75%) vaccines.

Vaccination delays observed in this study are similar or compare favourably with VD documented in other countries for MMR [13,14] or for vaccines containing certain elements of DTaP-Polio-Hib [30,31]. Nevertheless caution must be exercised in comparing VD data given the different methodologies employed.

Several reasons could explain the low impact of the intervention conducted herein. Firstly, this was an isolated intervention not combined with other types of interventional activities. Feedback that is integrated into other activities and sustained over time, such as that inspired by the Assessment, Feedback, Incentives and Exchange (AFIX) strategy, appears to be more likely to have an impact on vaccination [20,23,32]. Moreover, an increase in the number of doses administered in 2008-2009 was noted compared to 2007-2008. The pressure represented by this increased demand for vaccination could have contributed to the low impact of the feedback. As well, only half of the health professionals involved in vaccination in the clinics that were visited participated in the feedback process. The impact analyses, on the other hand, dealt with the totality of vaccination acts in the participating clinics, since the registry does not include a specific code for each vaccinator. Finally, VD presented were relatively low for some clinics and may not have generated sufficient motivation to change current practices. However, the feedback provided could have influenced more specific problematic practices resulting in high VD.

It is possible that the feedback was the cause of the decrease in VD observed for the four clinics that modified their practices concerning multiple injections. It is also possible that the introduction of the combined measles, mumps, rubella and varicella vaccine (MMR-V) administered at one year of age as well as the involvement of nurses in vaccination contributed to these changes. Two of the four clinics involved hired nurses or gave nurses additional responsibilities following the feedback. Other studies suggest that the involvement of nurses favours adherence to recommendations regarding vaccination [17]. These results are important, since the practice of multiple injections is a factor that facilitates complete immunization [33]. However, these modifications did not appear to have an impact on global analyses. A possible hypothesis is that the decrease in VD was observed in four of the smallest clinics that only gave a limited number of vaccine doses, therefore having a limited weight compared to larger clinics.

A relatively wide consensus exists concerning the definition of VD, namely one month after the date on the immunization schedule [34]. In the present study, the results using this standard did not suggest any deterioration or improvement in VD after feedback. When using a one-week standard, we observed an increase in VD for the first DTaP-Polio-Hib vaccine, for the first pneumococcal vaccine and for the meningococcal vaccine. However, these increases in VD correspond to a mean immunization time delayed by only one or two days in the year following the feedback, a difference not clinically significant. It is possible that using a one-week definition for VD may be too sensitive to very minor changes in immunization practices and lead to misinterpretations. Furthermore, using a short one-week standard for VD could demoralize clinicians because it is very difficult to reach. This issue was reported by some participants during the feedback sessions.

In Quebec, the Ministry of Health and Social Services tracks the vaccination delays associated with DTaP-Polio-Hib, pneumococcal and meningococcal vaccines. Strict surveillance of VD for DTaP-Polio-Hib vaccine is essential, as delays in its administration are associated with incomplete VC [3]. Since the VD for pneumococcal vaccine are almost identical to that for DTaP-Polio-Hib, and since it is scheduled for the same visit, the benefits associated with its surveillance appear rather limited.

A number of limitations of this study should be mentioned. A high proportion of vaccination acts are recorded in the vaccination registry used (VAXIN) [35]. However, when children move to the Quebec City region, it is possible that vaccines given previously in other regions may not be systematically reported and recorded. This phenomenon is apt to lead to an overestimation of VD, but should be consistent over time. The fact that the VD determined by this study are close to the data reported in provincial studies is nevertheless reassuring [3].

The questionnaire was completed in person after the first observation period and by telephone after the second observation period. As well, the same person was reached in only five of the ten clinics. An effort was made to limit the impact of this latter factor by formulating the questions in the same manner.

Finally, the use of a control group would have allowed for the impact of the feedback to be better identified. However, such a strategy would have been extremely difficult to apply since a large majority of doses are administered by a small number of clinics in the Quebec City region and the clinics that vaccinate the most were the ones that participated in the study. Nevertheless, we compared the VD observed during the two periods for those clinics that did not participate in the project and the same tendencies were noted.

## Conclusions

While the feedback that was provided did not lead to a decrease in VD, it nevertheless appeared to have facilitated certain positive changes in practices concerning multiple injections. It is possible that feedback integrated into other types of effective interventions, sustained over time or giving certain responsibilities to vaccinators may have more impact in decreasing VD. These measures, however, would require additional resources. Efforts aimed at reducing the burden related to delayed immunization and at improving VC should be pursued in order to protect the population against diseases that can be avoided by vaccination.

## Abbreviations

DTaP-Polio-Hib: Diphtheria, tetanus toxoid, acellular pertussis, inactivated poliovirus and *Haemophilus influenzae *type b vaccine; MMR: Measles, mumps and rubella vaccine; MMR-V: Measles, mumps, rubella and varicella vaccine; VC: Vaccine coverage; VD: Vaccination delays.

## Competing interests

Competing interests for CS: research grants, honoraria and reimbursement of travel costs by the following companies: Wyeth, GlaxoSmithKline and Merck Frost. The other authors declare that they have no competing interest.

## Authors' contributions

NB participated for the data collection and wrote the first draft of the manuscript. CS conceived the study, coordinated the data collection, carried out the feedback intervention, and helped in drafting the manuscript. MO and GD performed the statistical analysis and helped in drafting the manuscript. MK participated in data collection and helped in writing the first draft of the manuscript. DA, AP and CC prepared and conducted the feedback sessions for medical clinics, participated in data collection and helped in the writing of the manuscript. All authors read and approved the final manuscript.

## Pre-publication history

The pre-publication history for this paper can be accessed here:

http://www.biomedcentral.com/1471-2458/10/750/prepub
